# Trait Anxiety Does Not Predict the Anxiogenic Response to Sleep Deprivation

**DOI:** 10.3389/fnbeh.2022.880641

**Published:** 2022-07-04

**Authors:** Tina Sundelin, Benjamin C. Holding

**Affiliations:** ^1^Department of Psychology, Stockholm University, Stockholm, Sweden; ^2^Department of Clinical Neuroscience, Karolinska Institutet, Stockholm, Sweden; ^3^Department of Sociology, University of Copenhagen, Copenhagen, Denmark

**Keywords:** anxiety, STAI, sleep loss, individual differences, sleep restriction

## Abstract

Sleep deprivation has in several studies been found to increase anxiety. However, the extent to which this anxiogenic effect depends on one’s underlying trait anxiety has not previously been determined. Using two separate sleep-loss experiments, the current research investigated whether trait anxiety (STAI-T) moderates the increase in state anxiety (STAI-S) following one night of total sleep loss (study 1, *N* = 182, age 25.3 ± 6.5, 103 women) and two nights of partial sleep restriction (study 2, *N* = 67, age 26.5 ± 7.4, 38 women). Both studies showed the expected anxiogenic effect of sleep loss, and a clear relationship between trait anxiety and state anxiety. However, the anxiogenic effect of sleep loss was not moderated by trait anxiety, as there was an equal impact regardless of trait anxiety level. These findings indicate that, although sleep loss is related to general anxiety as well as anxiety disorders, for a non-clinical sample the anxiogenic effect of short-term sleep loss is not affected by baseline levels of anxiety.

## Introduction

Sleep is intertwined with daily anxiety, with higher levels of anxiety acting both as a predictor of sleep quality and a consequence of sleep loss (Alvaro et al., 2013; Horváth et al., 2016; Pires et al., 2016). Several studies have focused on the anxiogenic effect of sleep deprivation, suggesting that hypoactivity in the medial prefrontal cortex is a potential mechanism (Ben Simon et al., 2020a) and that gray matter volume may determine vulnerability differently for men and women (Goldstein-Piekarski et al., 2018), indicating that there are individual differences in this anxiogenic effect. Indeed, although the relationship between sleep and anxiety does not seem to depend on the impact of mood (Ben Simon et al., 2020a), the emotional responses to sleep loss of anxious individuals may be amplified compared to those with less trait anxiety (Goldstein et al., 2013; Alfano et al., 2020). This suggests that trait anxiety may predict vulnerability to the anxiogenic effects of sleep deprivation as well, but surprisingly little attention has been devoted to the relationship between trait anxiety and the effects of sleep loss in healthy subjects (Palmer and Alfano, 2020).

In order to understand who is vulnerable or resilient to the anxiogenic effects of sleep loss, more research is needed regarding underlying predictive factors. For example, there are large individual differences in how people respond to sleep deprivation in the cognitive domains (Tkachenko and Dinges, 2018), and these may in part be predicted by trait-level, domain-specific, cognitive abilities (Brieva et al., 2021; Floros et al., 2021). If the same is true for affective domains, those low in trait anxiety may have a smaller anxiogenic effect of sleep deprivation than those high in trait anxiety. For example, in adolescents with generalized anxiety disorder (GAD), natural variations in sleep duration has been shown as a predictor of morning anxiety, whereas for healthy controls it has not (Mullin et al., 2017). Specifically, for the adolescents with GAD, shorter sleep duration was associated with more morning anxiety the following day. However, as disturbed sleep is strongly comorbid with anxiety disorders it is difficult to disentangle cause and effect from observational studies of clinical samples. In other words, it is yet unknown whether there is a moderating effect of subclinical variations in trait anxiety on the anxiogenic effect of experimentally induced sleep loss. The aim of this brief report is thus to elucidate whether interindividual, subclinical variation in trait anxiety predicts the impact of sleep deprivation (study 1) and sleep restriction (study 2) on state anxiety.

## Methods

### Study 1

#### Participants

One hundred and eighty-two healthy participants were randomly assigned to one of two conditions: one night of total sleep deprivation (TSD; *N* = 91, average age 25.4 ± 6.21, range 18–45 years; 52 women) or normal 8h-sleep (NS; *N* = 91, average age 25.3 ± 6.82, range 17–45 years; 51 women). Exclusion criteria during the initial screening included physical or mental health problems, sleep disturbances, a subjective sleep need outside of 7–9 h, shift work in the previous 3 weeks, and addiction to coffee or other drugs (see Holding et al., 2019 for a complete list of exclusion criteria). Participants provided written informed consent and were compensated for their time. The study was approved by the Regional Ethical Review Board in Stockholm (2014/1766-21).

#### Procedure

Prior to being informed of their condition, participants filled out the trait version of the State-Trait Anxiety Inventory (STAI; Spielberger et al., 1983; Julian, 2011). This assesses “How you generally feel” with statements such as “I feel safe” and “I am a stable person.” The response options range from “1-almost never” to “4-almost always.” Participants were then instructed to sleep 8–9 h per night (between 22:00 and 08:00) in their home for three nights, with sleep times assessed using actigraphy (Sadeh, 2011) and sleep diaries. After the third night, they were informed of their sleep condition and were either instructed to spend one more night according to this schedule (NS group) and come into the lab at 10:00 the following day (the test day), or arrive at the lab at 22:00 that evening and stay awake until the following day (TSD group). Participants in the sleep-deprivation condition were monitored throughout the night, and not allowed to engage in any strenuous activities. Around 10:30 on the test day (∼26.5 h awake for the TSD group, ∼2.5 h awake for the NS group) they filled out the state version of STAI. This version assesses “How you feel at this moment” with statements such as “I feel safe” and “I feel nervous.” Response options range from “1-not at all” to “4-very much so.”

#### Analyses

State-Trait Anxiety Inventory was scored according to the standard instructions, adding up the responses to all items while reverse-scoring anxiety-absent items. This results in a value between 20 and 80 for each scale. All STAI scores were then converted to *z*-scores, and Bayesian linear modeling was used to test the main effects and interaction of TSD and trait anxiety on state anxiety. The Bayesian models provide a point effect estimate and a probability distribution of plausible alternative values (posterior distribution). To determine uncertainty around our point estimate, we use the 95% Credible Interval (95% CI; 2.5 and 97.5% quantiles of the posterior). The 95% credible interval is analogous to the 95% confidence interval though with greater precision (Gray et al., 2015).

A key benefit of taking a Bayesian approach is the ability to assess the relative strength of evidence for different hypotheses. Therefore, we can state (unlike in classical frequentist analysis which can only be used to reject the null hypothesis and never support it) whether the data supports the alternative hypothesis, the null hypothesis, or doesn’t provide enough information to support either. In other words, we can make clearer conclusions about situations in which no effect is present. In our case, we compare models with and without a coefficient of interest to calculate Bayes factors (BF_10_). A rule of thumb for interpreting these is that a BF_10_ greater than 3 suggests that there is evidence for the alternative hypothesis (i.e., that the coefficient improves the model prediction), a BF_10_ of less than 0.33 provides evidence of the null hypothesis (i.e., that the coefficients does not improve the model prediction), and a BF_10_ between these values suggests that there is not enough data to support either hypothesis (Dienes, 2014).

Data and analysis code can be found at https://osf.io/tcdne/. STAI values (both state and trait) over 3 SD from the mean were excluded. The data of three participants (all from the normal-sleep condition) were excluded for this reason. One further participant (from the normal-sleep condition) was excluded due to not completing the state STAI and another participant (from the sleep-deprivation condition) was excluded due to not completing the trait STAI.

The analysis was run using in R (version 4.1.0; R Core Team, 2021) with RStudio (version 1.4.1106; RStudio Team, 2021). To run the main analysis we used the brms (version 2.16.1; Bürkner, 2017) package using 8 chains and 40,000 iterations (20,000 warm-up). Priors were set on the coefficients with mean 0 and SD 1. All other parameters used the default non-informative regularizing priors of the brms package. To calculate the relative evidence of adding coefficients of interest (compared to a simpler model without this coefficient, i.e., the null hypothesis), we computed Bayes factors (BF_10_) using the BayestestR package (version 0.10.0; Makowski et al., 2019). For data preprocessing we used the following packages: dplyr, readxl, sjplot, tidyr, and readr (Wickham and Bryan, 2019; Wickham and Hester, 2020; Lüdecke, 2021; Wickham, 2021; Wickham et al., 2021).

### Study 2

#### Participants

Sixty-seven healthy participants (average age 26.5 ± 7.4, range 18–46 years; 38 women) took part in a within-subject protocol with two conditions: normal sleep and sleep restriction. Exclusion criteria included poor physical or mental health, sleep disturbances, a sleep need outside of 7–9 h, shift work during the previous 3 weeks, and an addiction to coffee or other drugs. Participants provided written informed consent and were compensated for their time. The study was approved by the Regional Ethical Review Board in Stockholm (2010/1506-31).

#### Procedure

Participants came to the lab on two occasions, with sleep conditions in a randomized counterbalanced order and a washout period of minimum 1 week between the two. They arrived at the lab in the afternoon (14:30 ± 1 h). The arrival time could vary between 13:00 and 16:00, but was always the same within participant. Upon arrival, they spent 30 min sitting down in a light-controlled room and filling out questionnaires. In the normal-sleep condition (two consecutive nights of 8 h of sleep) they filled out both the state and trait versions of STAI, and in the sleep-restriction condition (two consecutive nights of no more than 4 h of sleep) they filled out only the state version. In order to ensure adherence to the study protocol, participants wore actigraphs during all four nights, filled out sleep diaries, and sent a text message to the experimenter upon bedtime and waking.

#### Analyses

Hypotheses and analyses for study 2 were preregistered prior to looking at the data. All STAI scores were converted to *z*-scores, and Bayesian mixed-effect modeling was used to test the main effects and interaction of TSD and trait anxiety on state anxiety. Preregistration, data, and analysis code can be found at https://osf.io/ysa42 and https://osf.io/tcdne/.

As planned in the preregistration, STAI values (both state and trait) over 3 SD from the mean were excluded. Since the study was a repeated-measures cross-over design, it was possible for individuals to be excluded for a single session only. Two participants’ data were excluded in the sleep-restriction session for this reason, and thus their data is only represented in the normal-sleep session. A further participant did not complete the state STAI for the sleep-restriction session, and thus their data was only included in the normal-sleep session.

The statistical analysis was essentially identical to study 1. The only difference is that due to the repeated-measures design, we included random intercepts for participant ID to account for clustering of variance within these units.

## Results

See Table 1 for average sleep times and anxiety scores between groups and conditions. See Supplementary Table 1 for average sleep and wake times.

**TABLE 1:** Descriptive statistics.

	Condition	Sleep duration	State anxiety		Trait anxiety	
				
		Mean ± SD	Mean ± SD	Range	Mean ± SD	Range
Study 1	Normal sleep	471 ± 54	31.17 ± 7.11	20–54	37.51 ± 8.09	22–57
	Sleep deprivation	0 ± 0	35.22 ± 7.38	21–56	35.37 ± 7.10	22–57
Study 2	Normal sleep	466 ± 36	30.91 ± 7.58	20–55	34.01 ± 7.33	20–53
	Sleep restriction	245 ± 24	35.65 ± 7.01	23–55		20–53

*Study 1 is between participants, study 2 is within participants. Sleep duration is in minutes, based on actigraphy data. For study 1, it represents the last night before filling out the questionnaires; for study 2 it represents an average across the two nights before filling out the questionnaires. Data is missing for five participants in study 1 and for three participants in study 2 (see section “Methods” for more information).*

**FIGURE 1:**
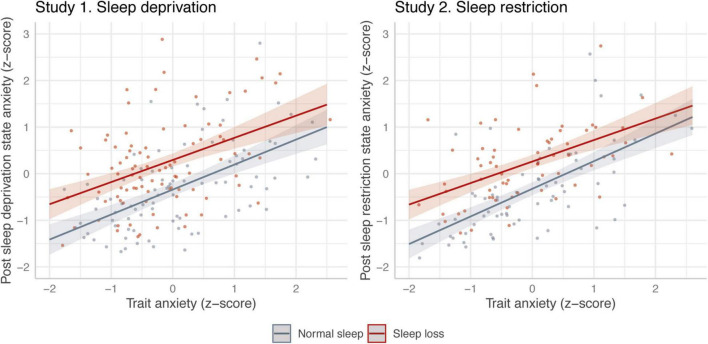
Association between trait anxiety and state anxiety reported before (gray) and after (red) sleep loss. The left-hand graph represents the results from study 1, using between-subjects sleep deprivation. The right-hand graph represents the results from study 2, using within-subjects sleep restriction. Model predictions are based on the models in Table 2. Solid line represents estimated association, with 95% credible intervals. Dots represent raw data points (jittered).

In both studies, sleep loss led to a clear increase in state anxiety, and trait anxiety predicted higher state anxiety for all groups and conditions (Table 2). However, there was no apparent moderating effect of trait anxiety on the anxiogenic effect of sleep loss (Table 2 and Figure 1). The Bayes factor of the interaction coefficients suggests that the data is approximately 4.6–6.8 times more likely to be generated under a model without a moderating effect of trait anxiety on the effect of sleep loss.

**TABLE 2:** Full models predicting state anxiety for studies 1 and 2.

	State anxiety (*z*-score)		
	
Predictors	Estimates	CI (95%)	BF_10_
**Study 1**			
Intercept	–0.34	−0.51, −0.17	
Sleep deprivation	0.64	0.40, 0.88	94.39
Trait anxiety (*z*-score)	0.54	0.36, 0.71	8540000000
Sleep deprivation × trait anxiety (*z*-score)	–0.06	−0.32, 0.20	0.148
**Study 2**			
Intercept	–0.32	−0.49, −0.16	
Sleep restriction	0.59	0.36, 0.81	3890
Trait anxiety (*z*-score)	0.59	0.42, 0.76	637000000
Sleep restriction × trait anxiety (*z*-score)	–0.13	−0.36, 0.10	0.219

*Study 1: observations = 177. R^2^ Bayes = 0.31. Study 2: random effects: ICC = 0.10. Observations = 133. Marginal R^2^ = 0.426. Conditional R^2^ = 0.492. BF_10_, Bayes factor for the alternative hypothesis, i.e., the model with the predictor added compared to the model without that predictor (i.e., the model with sleep restriction was compared to the intercept-only model, the model with trait anxiety was compared to the model with only sleep restriction, and the model with sleep restriction × trait anxiety was compared to the model with trait anxiety and sleep restriction).*

State anxiety when well rested and when sleep restricted were correlated (study 2; estimate = 0.46, 95% CI [0.23, 0.70], BF_10_ = 110.51 compared to intercept only model), and trait anxiety in a univariate model reliably predicted state anxiety (study 1: estimate = 0.46, 95% CI [0.32, 0.60], BF_10_ compared to intercept-only model = 42700000; study 2: estimate = 0.52, 95% CI [0.38, 0.66], BF_10_ compared to intercept-only model = 171000000).

As preregistered robustness checks, we ran the analysis in two further ways. Firstly, we compared participants in both studies who rated higher or lower than 1 SD from the mean trait anxiety score (see Supplementary Figure 1 and Supplementary Table 2). We also conducted the primary analyses without excluding any participants (i.e., not excluding outliers, see Supplementary Figure 2 and Supplementary Table 3). The results of these supplementary analyses follow the same pattern as the primary results presented.

As a non-preregistered robustness check, we also ran the analysis including participant gender as a covariate. The results follow the same pattern as the primary results presented (see Supplementary Table 4).

## Discussion

In the modern 24-society, sleep loss is prevalent (Webb and Agnew, 1975; Knutson et al., 2010; Shochat, 2012; Blom et al., 2020; but see Marshall and Lallukka, 2018 for discussion). Rates of anxiety are also high (Wiegner et al., 2015; Bosman et al., 2019), which studies have shown can have roots in lack of sleep (see Ben Simon et al., 2020b for review). We aimed to understand more about this relationship, by assessing whether the effect of sleep loss on anxiety was stronger in individuals who generally were already reporting high levels of anxiety.

In both studies, we found a clear influence of sleep loss on self-reported state anxiety. Sleep loss led to an increase in anxiety in the range of 0.60–0.65 SD, indicating a medium-to-large effect size (Sawilowsky, 2009). However, any potential moderation by trait anxiety of the impact of insufficient sleep on state anxiety was negligible. Bayes factors from both studies suggest that the data is much more likely if sleep loss has an approximately equal impact on state anxiety, irrespective of trait anxiety level. Additionally, this pattern was robust to two alternative methods of analyzing the data. These results provide important evidence regarding individual differences in the anxiogenic effects of sleep loss; although sleep loss causes an increase in state anxiety across levels of trait anxiety, the effect appears to be no different for those with high or low levels of trait anxiety.

The bidirectionality of sleep and anxiety has not been taken into account in this study. For example, it is possible that higher levels of anxiety following sleep loss is related to more subsequent rumination, in turn resulting in more troubled sleep (e.g., Gordon et al., 2019). This could amplify the effect for those high in trait anxiety across time, laying the foundation for the strong correlational relationship found between the two (e.g., Ben Simon et al., 2020a). Indeed, sleep disturbances in childhood are predictive of anxiety disorders in adulthood, even when controlling for internalizing problems and socioeconomic status (Gregory et al., 2005). For those who are more vulnerable, i.e., have higher trait anxiety, the increase in state anxiety due to sleep loss may thus be more detrimental even though it is not objectively larger. A potential limitation here is the use of a self-report measure of anxiety, which may be affected differently than more objective measures (see, e.g., Egloff and Schmukle, 2004).

Although higher trait anxiety seems to modify the emotional effects of sleep loss (Alfano et al., 2020; Palmer and Alfano, 2020), our results indicate that this is not due to differences in the anxiogenic effect of sleep loss. Rather, the resulting high levels of state anxiety for those higher in trait anxiety may be the explaining factor. It is possible that there is an inflection point at which one’s levels of anxiety relate to stronger emotional reactions to one’s environment.

The results of the two studies do not appear to be due to ceiling effects in state anxiety for those with high trait anxiety. The range of state anxiety was similar between the two groups (study 1) and two conditions (study 2), with no participant getting the maximum possible score in either. Although our exclusion of individuals with state anxiety over 3 SD above the mean may have inadvertently induced such a ceiling effect, especially in those with high trait anxiety, our supplementary analysis where we do not use this exclusion criteria (Supplementary Figure 2 and Supplementary Table 3) suggests that this did not affect the conclusions. Normative values for STAI-S in Sweden (where both studies were conducted) have been estimated at 33.2 ± 9.6, although this was based on a rather small sample (Forsberg and Björvell, 1993). Other reported normative values, in larger, non-Swedish samples were around 33–35.5 (SD ranging from 7.3 to 8.6) for STAI-S and 33–36 (SD ranging from 7.8 to 8.9) for STAI-T (Knight et al., 1983; Crawford et al., 2011). The participants in this study thus seem to represent a fairly normal population in terms of anxiety levels. Although those scoring high in trait anxiety (e.g., above 40 or 53) may be at a higher risk of developing an anxiety disorder (e.g., Dennis et al., 2013; Zingano et al., 2019), this was a subclinical sample and it is likely that individuals with clinical levels of anxiety respond differently to sleep loss, as indicated by previous research (e.g., Mullin et al., 2017).

In conclusion, variations in trait anxiety does not predict the anxiogenic effect of short-term sleep loss in a non-clinical sample. The findings of this study bring us closer to elucidating risk factors and protective mechanisms regarding the negative effects of sleep loss.

## Data Availability Statement

The datasets presented in this study can be found in online repositories. The names of the repository/repositories and accession number(s) can be found below: https://osf.io/tcdne/.

## Ethics Statement

The studies involving human participants were reviewed and approved by the Regional Ethical Review Board in Stockholm. The patients/participants provided their written informed consent to participate in this study.

## Author Contributions

TS and BH conceptualized and designed the study. TS organized the data collections and wrote the first draft on the manuscript. BH performed the statistical analyses. Both authors wrote sections of and revised the manuscript, and approved the submitted version.

## Conflict of Interest

The authors declare that the research was conducted in the absence of any commercial or financial relationships that could be construed as a potential conflict of interest.

## Publisher’s Note

All claims expressed in this article are solely those of the authors and do not necessarily represent those of their affiliated organizations, or those of the publisher, the editors and the reviewers. Any product that may be evaluated in this article, or claim that may be made by its manufacturer, is not guaranteed or endorsed by the publisher.
