# Supplementary material for: Trait Anxiety Does Not Predict the Anxiogenic Response to Sleep Deprivation
Source: Front Behav Neurosci. 2022 Jul 4;16:880641. doi: 10.3389/fnbeh.2022.880641 (PMC9332334; doi:10.3389/fnbeh.2022.880641)
Supplement: Supplementary file 1 [file Data_Sheet_1.docx]

Supplementary Material

# Supplementary Figures and Tables

**Table S1.** Average bed and wake times.

|  | **Normal sleep** | |  | | **Sleep loss** | | |
| --- | --- | --- | --- | --- | --- | --- | --- |
|  | Bedtime | Wake time | |  | | Bedtime | Wake time |
| Study 1 |  |  | |  | |  |  |
| Penultimate night | 23:42 | 07:34 | |  | | 23:43 | 07:24 |
| Experimental night | 23:45 | 07:37 | |  | | ­– | – |
| Study 2 | 23:47 | 07:31 | |  | | 01:35 | 05:40 |

**Table S2.** Robustness check models predicting state anxiety for studies 1 and 2 using high vs low levels of trait anxiety.

|  | **State anxiety (z-score)** | | |
| --- | --- | --- | --- |
| *Predictors* | *Estimates* | *CI (95%)* | *BF_10_* |
| Study 1 |  |  |  |
| Intercept | -0.89 | -1.38,  -0.38 |  |
| Sleep deprivation | 0.57 | -0.10,  1.23 | 0.936 |
| High Trait anxiety | 1.28 | 0.65,  1.89 | 23100 |
| Sleep deprivation x High trait anxiety | 0.17 | -0.69,  1.04 | 0.471 |
| Study 2 |  |  |  |
| Intercept | -0.98 | -1.49,  -0.45 | -0.98 |
| Sleep restriction | 0.51 | -0.15,  1.14 | 0.51 |
| High Trait anxiety | 1.63 | 0.91,  2.29 | 1.63 |
| Sleep restriction x High trait anxiety | -0.28 | -1.13,  0.63 | -0.28 |
| Note. Study 1: Observations = 47. R^2^ Bayes = 0.432. Study 2: Random effects: ICC = 0.11. Observations = 40. Marginal R^2^ = 0.486. Conditional R^2^ = 0.550.  BF_10_ = Bayes factor for the alternative hypothesis, i.e. the model with the predictor added compared to the model without that predictor (i.e. the model with sleep restriction was compared to the intercept-only model, the model with trait anxiety was compared to the model with only sleep restriction, and the model with sleep restriction x trait anxiety was compared to the model with trait anxiety and sleep restriction).  **Table S3**. Robustness check models predicting state anxiety for studies 1 and 2 without removing outliers.   \|  \| **State anxiety (z-score)** \| \| \| \| --- \| --- \| --- \| --- \| \| *Predictors* \| *Estimates* \| *CI (95%)* \| *BF_10_* \| \| Study 1 \|  \|  \|  \| \| Intercept \| -0.31 \| -0.48,  -0.13 \|  \| \| Sleep deprivation \| 0.60 \| 0.35,  0.85 \| 10.35 \| \| High Trait anxiety \| 0.55 \| 0.39 , 0.71 \| 192000000000 \| \| Sleep deprivation x High trait anxiety \| -0.07 \| -0.33,  0.18 \| 0.152 \| \| Study 2 \|  \|  \|  \| \| Intercept \| -0.32 \| -0.50,  -0.14 \|  \| \| Sleep restriction \| 0.65 \| 0.41,  0.90 \| 12000 \| \| High Trait anxiety \| 0.59 \| 0.41,  0.77 \| 13000000000 \| \| Sleep restriction x High trait anxiety \| -0.01 \| -0.26,  0.23 \| 0.124 \| \| Note. Study 1: Observations = 180. R^2^ Bayes = 0.314. Study 2: Random effects: ICC = 0.07. Observations = 135. Marginal R^2^ = 0.453. Conditional R^2^ = 0.493.  BF_10_ = Bayes factor for the alternative hypothesis, i.e. the model with the predictor added compared to the model without that predictor (i.e. the model with sleep restriction was compared to the intercept-only model, the model with trait anxiety was compared to the model with only sleep restriction, and the model with sleep restriction x trait anxiety was compared to the model with trait anxiety and sleep restriction).  **Table S4**. Robustness check models predicting state anxiety for studies 1 and 2 including gender as a covariate.   \|  \| **State anxiety (z-score)** \| \| \| \| --- \| --- \| --- \| --- \| \| *Predictors* \| *Estimates* \| *CI (95%)* \| *BF_10_* \| \| **Study 1** \|  \|  \|  \| \| Intercept \| -0.28 \| -0.49 – -0.06 \|  \| \| Female \| -0.11 \| -0.35 – 0.13 \| 0.185 \| \| Sleep deprivation \| 0.64 \| 0.40 – 0.88 \| 99.90 \| \| Trait anxiety (z-score) \| 0.54 \| 0.36 – 0.71 \| 8280000000 \| \| Sleep deprivation x Trait anxiety (z-score) \| -0.06 \| -0.32 – 0.20 \| 0.147 \| \| **Study 2** \|  \|  \|  \| \| Intercept \| -0.36 \| -0.58 – -0.13 \|  \| \| Female \| 0.06 \| -0.20 – 0.32 \| 0.235 \| \| Sleep restriction \| 0.58 \| 0.36 – 0.81 \| 3930 \| \| Trait anxiety (z-score) \| 0.59 \| 0.42 – 0.76 \| 386000000 \| \| Sleep restriction x Trait anxiety (z-score) \| -0.13 \| -0.36 – 0.10 \| 0.223 \| \| Note. Study 1: Observations = 177. R^2^ Bayes = 0.32. Study 2: Random effects: ICC = 0.11. Observations = 133. Marginal R^2^ = 0.428. Conditional R^2^ = 0.495.  BF_10_ = Bayes factor for the alternative hypothesis, i.e. the model with the predictor added compared to the model without that predictor (i.e. the model with sleep restriction was compared to the intercept-only model, the model with trait anxiety was compared to the model with only sleep restriction, and the model with sleep restriction x trait anxiety was compared to the model with trait anxiety and sleep restriction). \| \| \| \| \| \| \| \| | | | |

**Figure S1**. Association between trait anxiety and state anxiety reported after normal sleep (grey) and after sleep loss (red). The left-hand graph represents the results from study 1, using between-subjects sleep deprivation. The right-hand graph represents the results from study 2, using within-subjects sleep restriction. Low trait anxiety represents individuals 1SD or more under the mean. High trait anxiety represents individuals 1SD or more above the mean. Model predictions are based on the models in tables S1. Solid line represents estimated association, with 95% credible intervals. Dots represent raw data points (jittered).

**Figure S2**. Association between trait anxiety and state anxiety reported before (grey) and after (red) sleep loss, with no outliers removed. The left-hand graph represents the results from study 1, using between-subjects sleep deprivation. The right-hand graph represents the results from study 2, using within-subjects sleep restriction. Model predictions are based on the models in tables 2. Solid line represents estimated association, with 95% credible intervals. Dots represent raw data points (jittered).
